# Microfibers synthesized by wet-spinning of chitin nanomaterials: mechanical, structural and cell proliferation properties†

**DOI:** 10.1039/d0ra06178f

**Published:** 2020-08-10

**Authors:** Ling Wang, Nazanin Zanjanizadeh Ezazi, Liang Liu, Rubina Ajdary, Wenchao Xiang, Maryam Borghei, Hélder A. Santos, Orlando J. Rojas

**Affiliations:** Department of Bioproducts and Biosystems, Aalto University P. O. Box 16300 00076 Aalto Finland orlando.rojas@ubc.ca; Drug Research Program, Division of Pharmaceutical Chemistry and Technology, Faculty of Pharmacy, University of Helsinki FI 00014 Helsinki Finland; College of Chemical Engineering, Nanjing Forestry University Nanjing 210037 China; Helsinki Institute of Life Science (HiLIFE), University of Helsinki FI 00014 Helsinki Finland; Bioproducts Institute, Department of Chemical and Biological Engineering, Department of Chemistry and Wood Science, University of British Columbia 2360 East Mall Vancouver V6T 1Z3 BC Canada

## Abstract

Partially deacetylated chitin nanofibers (ChNF) were isolated from shell residues derived from crab biomass and used to prepare hydrogels, which were easily transformed into continuous microfibers by wet-spinning. We investigated the effect of ChNF solid content, extrusion rate and coagulant type, which included organic (acetone) and alkaline (NaOH and ammonia) solutions, on wet spinning. The properties of the microfibers and associated phenomena were assessed by tensile strength, quartz crystal microgravimetry, dynamic vapor sorption (DVS), thermogravimetric analysis and wide-angle X-ray scattering (WAXS). The as-spun microfibers (14 GPa stiffness) comprised hierarchical structures with fibrils aligned in the lateral direction. The microfibers exhibited a remarkable water sorption capacity (up to 22 g g^−1^), while being stable in the wet state (50% of dry strength), which warrants consideration as biobased absorbent systems. In addition, according to cell proliferation and viability of rat cardiac myoblast H9c2 and mouse bone osteoblast K7M2, the wet-spun ChNF microfibers showed excellent results and can be considered as fully safe for biomedical uses, such as in sutures, wound healing patches and cell culturing.

## Abstract 2

Chitin nanomaterials are wet-spun into microfibers that are biocompatible and show promise for their cell viability and proliferation.

## Graphical abstract

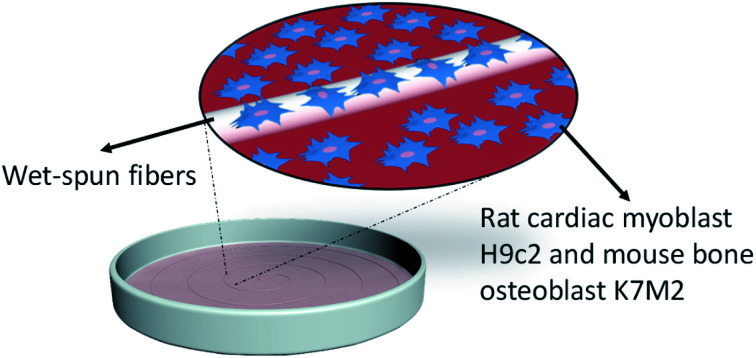

## Introduction

Chitin is well-known for its renewability, nontoxicity, biodegradability, biocompatibility, and antibacterial properties. It is mainly sourced from ocean biomass as well as fungi and algae.^1–3^ The potential availability of chitin is illustrated by the 6 to 20 million tons of crab, shrimp and lobster shells processed every year.^1,4^ Such residual ocean biomass is usually landfilled or directly dumped in coastal areas. Transforming such an abundant resource into valuable products has become a critical need. Meanwhile, chitin can be suitably processed into different shapes, namely, fibers, films, and aero/hydrogels. Chitin fibers have attracted increased interest given their high surface area and facile processability, which can be further expanded in the fabrication of 2D- and 3D structures.

Though various techniques have been explored for chitin spinning, the general rules are the same: a harsh, non-green solvent is necessary in order to break down the hydrogen bonds in the material and to dissolve the molecules, after which a coagulant or anti-solvent can be used to recrystallize chitin.^5,6^ The solvent can be an ionic liquid, *N*,*N*-dimethylacetamide/LiCl, CaCl_2_/methanol, alkali/urea.^1^ A reasonable alternative, however is to directly exploit the nanostructure of chitin in the design and development of high-order constructs while minimizing or avoiding dissolution (and subsequent regeneration). Indeed, nature has optimized the nanocrystalline dimensions and morphology of chitin, for an excellent combination of stiffness, strength, and toughness.^5^

To this end, wet-spinning has been applied to assemble nanostructures into one-dimensional fibers, for example, those from cellulose nanofibrils (CNF). After adjusting the spinning conditions, super-strong CNF fibers have been manufactured,^7^ which compete with the properties of known natural or synthetic biopolymeric materials. Moreover, owing to their abundant surface hydroxyl groups, functional fibers (*e.g.*, magnetic,^8^ superabsorbents,^9^ water-resistant,^10^ specific polymer sorption^11^) they can be obtained by optimizing the spinning dope, coagulant, and post-treatment.

Chitin nanofibrils (ChNF), which have a similar structure as that of CNF, offer potential as a precursor for wet-spinning. However, this is a topic that has been studied only to a limited extent. The few reports available in this area include that of Walther *et al.* who conducted wet spinning of ChNF using tetrahydrofuran as coagulation medium. The main focus of this latter work was to study the effect of stretching on the mechanical properties of the obtained fibers.^12^ Recently, we reported on the production of composite fibers from ChNF and alginate by complexation during dry spinning.^13^

From the biological perspective, ChNF is attractive in functional foods, to manage cholesterol levels and body weight. For instance, several *in vitro* and *in vivo* studies have indicated that chitin-based polymers can inhibit lipid digestion or absorption given that it strongly binds to bile acids.^14–16^ Moreover, ChNF has been shown to protect skin cells by improving the epithelial granular layer and increasing granular density.^17^ The clinical and histological assessment demonstrated ChNF to reduce skin inflammation (atopic dermatitis),^18^ and was associated with the production of collagen fibers.^19^ Recently, ChNF-based products have been commercialized for uses in the food industry, cosmetics and for skin and wound treatments. In addition, owing to its biocompatibility, easy processing into different shapes and structures as well as biodegradability, chitin has been reported as one of the preferred candidates for drug delivery,^20^ biomaterial science and tissue engineering.^21–23^ Chitin undergoes biodegradation by lysozyme and chitinase enzymes and by macrophages *in vivo*,^24^ making it functional for tissue engineering. Calcium phosphate/chitin nanofiber hydrogel as an inorganic/organic nanocomposite, for example, was investigated for bone application *in vivo*, showing the biocompatibility of the system and mineralization induction using Sprague Dawley rats.^25^

Manufacturing chitin into fibers would expand its application in both skin and tissue engineering since it can be further knitted into 2D and 3D shapes. Chitin-based fibrous systems introduced considerable improvement in the healing process in single or composite matrices.^22^ Noh *et al.*, for example, demonstrated a positive interaction between electrospun chitin nanofibers coated with type I collagen and human keratinocytes and fibroblasts.^26^ Recently, Zhu *et al.* have investigated the myocyte attachment and beating on the chitin fibers in fibrinogen/Matrigel hydrogel made by solution spinning, showing a great potential of chitin in cardiac applications.^6^ Additional relevance to the medical field is in the manufacture of sutures, which become in contact with different tissues present in the biological environment, such as bone and tendon, heart muscle in cardiac surgeries and skin in wound healing.^27,28^

In sum, the results so far indicate that chitin offers promise as a microfiber structure and have a great potential for surgical suturing and tissue healing. Thus, in this work, we introduce wet-spun chitin microfibers. For this, chitin was first isolated from fresh crab shells and then partially deacetylated and microfluidized into nanofibrils (ChNF). ChNF in aqueous suspensions of different concentrations were directly spun into a coagulation bath, either organic (acetone) or alkaline (NaOH and ammonia). Microfibers were successfully produced and systematically characterized in terms of morphology, mechanical strength (dry and wet state), water/moisture sorption capacity, and thermostability. The biocompatibility and cell proliferation were investigated *in vitro* using myoblasts and osteoblasts cells from cardiac muscle and bone tissue, respectively.

## Experimental

### Isolation of chitin nanofibrils (ChNF)

Alpha (α)-chitin nanofibrils were produced following our previous report.^29^ In short, the shells of cooked crabs were used as precursors, which were purified three times by consecutive immersion in 1 M HCl and 1 M NaOH for 12 h. Further, decoloration was carried out by using 0.5% (w/w) NaClO_2_ at pH 5 for 2 h at 70 °C. After rinsing with distilled water, the purified chitin was crushed into small sizes (household blender) and deacetylated using 33% (w/w) NaOH solution at 90 °C for 4 h. The partially deacetylated chitin was washed with distilled water and passed through a microfluidizer (M-110P, Microfluidics IN., Newton, MA, USA) using 400 and 200 μm chambers at 1500 bar. Finally, the obtained (partially deacetylated) chitin nanofibrils were titrated to pH 3.5 using acetic acid. *ζ*-Potential was measured with 5 ml 0.05% ChNF suspension using Zetasizer Nano (ZS-90, Malvern Instruments).

### Apparent degree of deacetylation (DD)

The apparent degree of deacetylation of ChNF was 19.5 ± 1.35, as determined by elemental analysis (PerkinElmer 2400 CHNS/O Elemental Analyzer) *via*eqn (1).^30^1
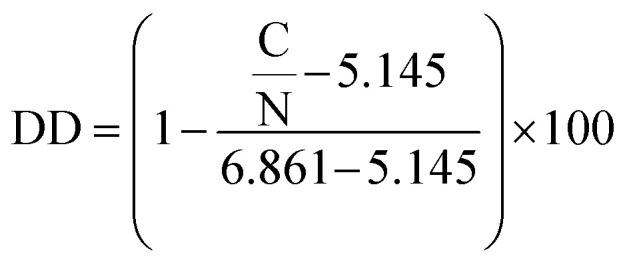
where C/N is the measured carbon to nitrogen ratio, 5.145 is the C/N ratio of completely deacetylated chitin, 6.861 is the C/N ratio of fully acetylated chitin. Note that ChNF were first freeze-dried and then oven-dried at 120 °C for 2 h to evaporate the excess acetic acid before the measurements.

### ChNF morphology

Transmission electron microscopy (TEM, JEM-2800, JEOL, Japan) and atomic force microscopy (AFM, Nanoscope V controller, Bruker Corporation, USA) were used to study the morphology of ChNF. To prepare the TEM samples, a drop of diluted ChNF suspension (0.005%) was deposited on a plasma-cleaned holey carbon grid. After drying, the images were recorded with a Gatan CCD camera. TEM observation was conducted at an acceleration voltage of 120 kV. To prepare the AFM specimens, diluted ChNF was spin-coated on mica and dried in air. The AFM imaging was carried in air, operated in tapping mode and using a J-scanner.

### ChNF microfiber synthesis

ChNF suspended in water at given various concentration (0.5, 1, 1.5 and 2 wt%) was loaded into a syringe and extruded through a tubing (length 44.5 cm, inner diameter 6 mm), and finally into acetone by using a needle (0.7 × 40 mm). Two extrusion rates (1 and 2.6 m min^−1^) were applied during spinning. The microfibers (F) obtained from acetone (AC) coagulation using a low rate (L, 1 m min^−1^) are referred to by adding the corresponding letter code together with the concentration, namely, F_AC_0.5_L, F_AC_1_L, F_AC_1.5_L, F_AC_2_L. The corresponding microfibers spun at the higher rates (2.6 m min^−1^) were named as F_AC_0.5, F_AC_1, F_AC_1.5, F_AC_2. In addition, 2 wt% ChNF was extruded through either ammonium or sodium hydroxide solutions (0.5 M), at a spinning rate of 2.6 m min^−1^, obtaining microfibers F_AM_ and F_Na_, respectively. In order to study the effect of coagulant concentration on microfiber properties, NaOH solutions at a concentration of 0.001 M (pH 11), 0.5 M, and 1 M as well as ammonium hydroxide at 0.55 M (pH 11) were applied using wet spinning of 2 wt% ChNF at 1 m min^−1^. The microfibers were labelled as F_Na_0.5M_L, F_Na_1M_L, and F_AM_ pH11_L. The microfibers were collected as soon as they formed. Those from ammonium hydroxide and NaOH coagulation were washed with water before drying. All microfibers were dried under tension at room temperature.

### Quartz crystal microbalance with dissipation (QCM-D)

A Q-sense E4 apparatus (Q-sense, Sweden) was utilized to study the interaction of ChNF with the given coagulants (ethanol, 0.5 M NaOH, and 0.5 M ammonia). The experiments were conducted at 23 °C and under a constant flow of 200 μl min^−1^. To prepare the surfaces used in QCM experiments, ChNF at 0.005 wt% concentration was spin-coated on silica crystals and dried under nitrogen flow. The crystals coated with ChNF were rinsed with degassed MilliQ water and placed in the QCM unit in contact with water until the frequency and dissipation signals reach stable values. After 12 min, degassed ethanol or aqueous solutions (0.5 M NaOH or ammonium hydroxide) were introduced for 90 and 360 min, respectively. Subsequently, the films were rinsed with degassed MilliQ water. Note: since acetone swells the QCM O-ring, ethanol was used instead.

### ChNF microfiber morphology

The morphology of ChNF microfibers was accessed by scanning electron microscopy (SEM) using Zeiss SIGMA VP (Carl Zeiss Microscopy Ltd, Cambridge, UK) operated at a working distance of 1 cm at 1.6 kV. Prior to imaging, the microfibers were sputter-coated with 5 nm Pt/Pd using a sputter coater (LECIA EM ACE600).

### Mechanical strength of ChNF microfibers

The stiffness and strength of the wet-spun microfibers were determined by using an Instron 5944 Single Column, Tabletop Universal Testing System operated in tensile mode with a load cell of 5 N. Before testing, all the microfibers were conditioned overnight at 50% relative humidity (RH) and 23 °C. The cross-sectional areas were obtained *via* SEM and software ImageJ. Seven specimens from each sample were tested by using a strain rate of 2 mm min^−1^ and a gauge length of 20 mm.

The linear fiber density (titer) as well as dry and wet tenacities were measured based on ISO 5079 standard, at room temperature and 65% RH using Vibroskop 400 and Vibrodyn 400 (Lenzing Instruments GmbH & Co KG, Austria). Before testing, the microfibers were conditioned overnight at 65% RH. The tenacity was measured using a gauge length of 20 mm, strain rate of 20 mm min^−1^, and a preload of 2000 mg. Ten specimens of each microfiber were measured.

### Microfiber interactions with water

The amount of water absorbed by the microfibers was determined using dry microfibers of known weight. They were placed in a teabag, immersed in water for 60 min, drained, and weighted again after 10 min. Three blank teabags were also run and the mass of water absorbed by each gram of teabag was determined. The effective mass of water absorbed by the samples was calculated using the following equation:
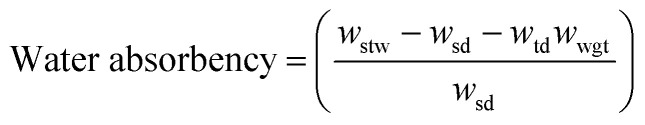
where *w*_stw_ is the wet weight of the ChNF microfibers and teabag, *w*_sd_ is the weight of dry ChNF microfibers, *w*_td_ and *w*_wgt_ are the teabag dry weight and the water absorbed per gram of teabag, respectively.

The water vapor sorption capacity of the spun microfibers was determined using dynamic vapor sorption (DVS, Intrinsic apparatus, Surface Measurement Systems, UK) with an accuracy of 0.1 μg. About 5 mg of microfibers were cut and loaded in the chamber of the microbalance with controlled relative humidity (RH). Before testing, the microfibers were preconditioned until equilibration at 0% RH. Then, the RH was cycled between 0% and 95% for seven times at room temperature and under nitrogen atmosphere. The moisture sorption capacity was determined from the mass changes.

### Cell culturing *in vitro*

Cardiac myoblast H9c2 (ATCC® CRL-1446™) and mouse bone osteoblast K7M2 [K7M2-WT] (ATCC® CRL-2836™) were cultured in cell culture flasks of Dulbecco's Modified Eagle's Medium (DMEM) supplemented by 10% of fetal bovine serum (FBS), 1% (w/v) of l-glutamine, 1% (w/v) of non-essential amino acids (NEAA), and penicillin–streptomycin (100 IU ml^−1^) at 37 °C in 5% CO_2_. Cell medium was changed every two days until reaching 80% of cell confluence before starting the test.

### Biocompatibility and cell proliferation

The proliferation of the rat cardiac myoblast H9c2 and mouse bone osteoblast K7M2 in the vicinity of the three types of wet-spun microfibers (F_AC_, F_Na_, F_AM_) was investigated. About 2 mg of each microfiber were sterilized by first ethanol immersion and then drying under UV-light for 3 h. Samples were immersed inside DMEM + 10% FBS and antibiotics for 24 h. About 10 000 cells of heart and bone were counted and seeded in each sample well in 24-well plates (four replicates) and cell wells without microfibers were used as the positive control (+control). In each time point (day 1 and 7), the medium was soaked out and AlamarBlue solution in pure DMEM was added to each well. The formed fluorescent resorufin was collected and inserted in new 96-well plates and tested by Varioskan Flash plate reader (Thermo Scientific Inc.) to check the cell growth and viability.

## Results and discussion

### ChNF fibril morphology

Partially deacetylated chitin (deacetylation degree of 19.5 ± 1.3) was defibrillated into nanofibrils *via* microfluidization and stored in acidic conditions (pH 3.5). The acetic acid addition simultaneously achieved partial shifting of *N*-acetyl groups to protonated amino groups. As a result, a positively charged ChNF was obtained with a *ζ*-potential value of 50 ± 2 mV.

**Fig. 1:**
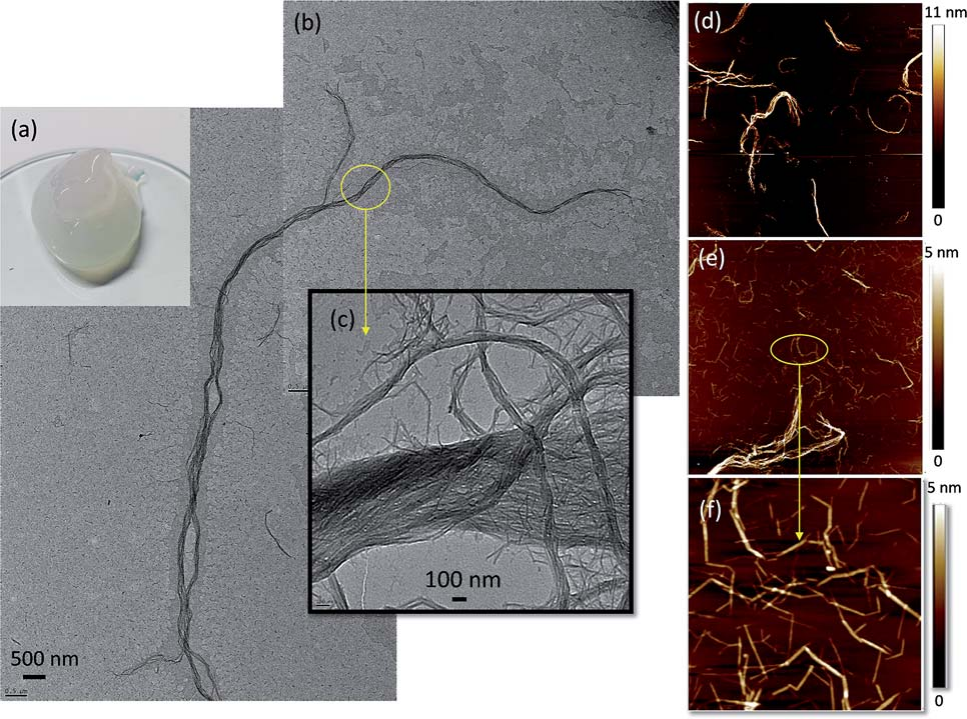
(a) Visual appearance, (b and c) TEM micrograph and ((d) 20 × 20 μm; (e) 3 × 3 μm; (f) 1 × 1 μm) AFM images of as-prepared ChNF.

As shown in Fig. 1a, the partially deacetylated chitin nanofibers, thereafter termed “ChNF”, showed a semi-transparent, gel-like appearance in aqueous suspension. Both TEM (Fig. 1b) and AFM (Fig. 1d) micrographs indicated that, after mechanical disintegration, chitin was successfully deconstructed into nanofibrils. The TEM images further show the high axial aspect ratio of the fibrils. As shown in Fig. 1b, the obtained ChNF possesses a diameter about 200 nm, while the length was of the order of tens of micrometers. The magnified image (Fig. 1c) shows that fibrils contained loosely bound, bundled nanofibrils. Similar observation was made in the preparation of chitin nanocrystals.^31^ Positively charged amino groups facilitate the defibrillation of chitin fibrils.^32^ Since amino groups are randomly distributed on the surface, a lateral disassembly occurs during the mechanical process (microfluidization). Besides, the interactions (*e.g.*, hydrogen bonding) between the surface functional groups of chitin and surrounding water^33^ lead to swelled/loosen chitin bundles given that water can penetrate in the deacetylated chitin.^31^

The presence of fibril bundles was also observed in AFM images (Fig. S1†). Further AFM imaging revealed a heterogeneous fibril size (Fig. 1d and e). Since the lateral disassembly occurred from the surface to the inner regions, the surface with more amino groups would be easier to defibrillate, hence shorter fibrils were obtained. With the severity of mechanical treatment, the chitin surface was defibrillated, exposing the larger, inner fibrils. Moreover, the small fibrils were connected end-to-end (Fig. 1f), likely caused by inefficient defibrillation. Since the deacetylation occurs non-uniformly on the surface of chitin, those positions with more amino groups were easier to defibrillate, leaving very thin fibrils connected with the regions containing less amino groups.

### ChNF microfiber formation

Aqueous ChNF suspensions of different concentration (0.5, 1, 1.5, 2 wt%) were extruded using an acetone bath. During coagulation, a counter solvent exchange occurred between water in ChNF and the coagulant (acetone). Consequently, ChNF lost water, underwent phase separation, and settled down, at the bottom of the bath, given the densification process.^34^ After coagulation, the microfiber required quick withdrawal from the coagulation bath since it would otherwise stick to the bottom surface (*e.g.*, glass, metal or plastic). For diluted ChNF suspensions, a longer coagulation time was needed and the formed microfibers adhered more strongly to the bottom surface of the bath. All suspensions were spun into microfibers except the one at 0.5 wt% concentration, given the insufficient fibril interactions.

**Fig. 2:**
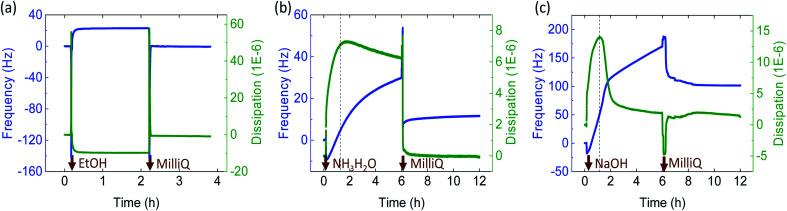
Shift in frequency (blue profiles) and dissipation (green profiles) of ChNF-coated QCM-D crystals as a function of time upon contact with (a) an organic solvent (ethanol, EtOH), (b) ammonia and, (c) NaOH solution. Note: ethanol was used instead of acetone to avoid interference from O-ring dissolution.

In order to investigate the coagulation mechanism, ChNF-coated QCM-D crystals were exposed to the coagulants (Fig. 2). As shown in Fig. 2a, introduction of ethanol to ChNF, caused a rapid increase in frequency (+23 Hz, indicating a mass loss) and a simultaneous reduction of dissipation (16 × 10^−6^). Both results indicate a loss of associated water, displaced by the organic solvent as well as densification. The decline in dissipation indicated that the film became more “rigid”. This is in line with the observation that a semi-solidified microfiber was formed in contact with the organic coagulant. Upon subsequent exposure to water, the ChNF returned to the original state. Similar behavior was reported for negatively charged CNF (TOCNF).^34^

An aqueous alkaline electrolyte (NaOH and ammonia) solution was applied as coagulants. When the positively charged ChNF was extruded in alkaline solution, the primary protonated amine groups were neutralized (from NH_3_^+^ to NH_2_). This resulted in a decrease in ionic repulsion, leading to loss of water from ChNF and cross-linking *via* hydrogen bonding and hydrophobic interactions.^35,36^ In the end, hydrogel microfibers were formed and became less dense (they were drifted to the surface of the coagulant bath). QCM-D experiments demonstrated (Fig. 2b and c) that as the given solution (ammonia or NaOH) was introduced onto the ChNF-coated sensors, the frequency increased rapidly (+20 Hz after 2 h and +50 Hz after 1.1 h, respectively). This is explained by the deprotonation of amino groups and the release of excess water. Meanwhile, the dissipation increased (7 × 10^−6^ at 2 h for ammonia and 14 × 10^−6^ at 1.1 h for NaOH) indicating that the ChNF layer became “softer” and more “viscous”, in agreement with results observed for the hydrogel microfibers formed in the alkaline coagulation baths. However, with time, the frequency increased and dissipation decreased (30 Hz and 6 × 10^−6^ for ammonia, 171 Hz and 2 × 10^−6^ for NaOH at 6 h) (see Fig. 2b and c, right side of the dash line). Compared to ammonia, NaOH had a greater impact on both frequency and dissipation. During this phase, ChNF deacetylation, partial dissolution as well as desorption may have occurred, which agree with observations made for the microfibers. Subsequently, by changing the medium back to water, the frequency remained higher than that of the original state (11 Hz for ammonia, 101 Hz for NaOH). It is likely that the alkaline solutions gradually interfered with the ionic bonding between ChNF and silica crystal, as the ions diffused deeper into the ChNF film. Consequently, a dramatic and continuous increase of frequency occurred in both alkaline conditions. To validate whether the running media contributed to the gain of frequency, NaOH solution was kept in contact with the crystals, (with no circulation): similar phenomena were observed, with a less increased frequency (Fig. S2†), indicating that desorption occurred between ChNF and silica crystals (also, flow or shear-enhanced desorption).

For wet-spinning, alkaline solutions of different concentrations were applied as coagulants. NaOH at 0.5 and 1 M and ammonia at 0.5 M and 0.55 M (pH 11) were effective to coagulate ChNF into microfibers. Interestingly, when NaOH at pH 11 (0.001 M) was applied, no microfibers were formed. In contrast, ammonia solution successfully coagulated microfibers (note: the ammonia concentration is much higher than that of NaOH at the same pH).

### Fiber morphology and fibril orientation

**Fig. 3:**
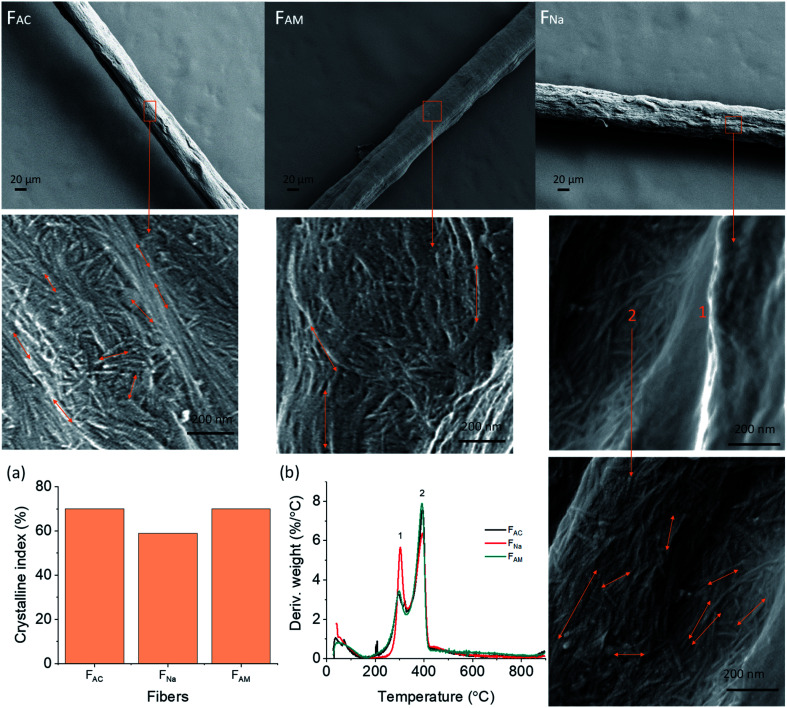
SEM images of the surface of microfibers observed at low and high magnification. (a) Crystalline index as well as (b) differential thermogravimetric (DTG) profiles of wet-spun ChNF microfibers obtained using given coagulants.

SEM micrographs demonstrate the morphology of the microfibers on the surface and cross-sections (Fig. 3). Microfibers coagulated in acetone (F_AC_), and ammonia (F_AM_) had a smoother surface than the ones spun in NaOH (F_Na_) which were covered with some layers (Fig. S3†). The F_AC_ (see magnified images in Fig. 3) showed both long and short needle-like nanofibrils on the surface, which were hardly observed in F_Na_. F_Na_ that displayed smooth surfaces (high magnification image, see “1” in Fig. 3). However, few parts of the microfiber remained intact, with a fibrillar structure (Fig. 3). On the other hand, the surface of F_AM_ contained larger nanofibrils, partially merged together, indicating that fibrils swelled during coagulation. In all cases, the nanofibrils tended to align in the lateral direction, with some randomly distributed needle-like crystals. This observation is somewhat in contrast to microfibers obtained from cellulose nanofibers (CNF) or those from chitin/alginate, which display randomly distributed nanofibrils.^13^ Nanofibrils align under shear, while Brownian motion impacts alignment negatively.^37^ Thus, we hypothesize that microfibers of low aspect ratio could lead to fast relaxation. In this study, since ChNF contained fibrils with a high aspect ratio (Fig. 1), the spun microfibers showed the tendency for ChNF to align along the lateral direction. The fibril orientation index as well as Herman's parameter were determined by wide-angle X-ray scattering, WAXS (Fig. S4,† an index of 1 correspond to fully aligned structures while “0” indicates random orientation). Similar fibril orientation was observed for all microfibers, whereas microfibers coagulated in alkaline solution (F_Na_ and F_AM_) showed slightly higher fibril orientation (orientation index of 0.78 and 0.74 and Herman's parameter of 0.59 and 0.61, respectively) compared to F_AC_ (0.72 and 0.54, respectively).

To determine whether partial dissolution and regeneration occurred for chitin microfibers during coagulation, the wet-spun microfibers were characterized for crystallinity and thermal stability. The regenerated chitin is supposed to present less crystallinity.^6^ Accordingly, WAXS was applied to detect chitin allomorphs as well as crystallinity degree. All the microfibers remained as α-chitin, as indicated by the four sharp crystalline reflections at 2*θ* = 9.5, 12.8, 19.5, and 26.5 in Fig. S5.† The microfiber crystalline degree was calculated in terms of crystalline index (CI), according to the following expression:^38^CI(%) = [(*I*_110_ − *I*_am_)/*I*_110_]×100where *I*_110_ is the maximum scattering intensity at the (110) plane at 2*θ* = 19° and *I*_am_ is the scattering intensity of the amorphous region in the same unit (101 plane) at 2*θ* = 12.6°.

Accordingly, F_AC_ and F_AM_ showed 70% crystalline index, higher than that of F_Na_ (59%) (Fig. 3a). Thus, F_Na_ (coagulated in 0.5 M NaOH), likely underwent dissolution-regeneration. This is in contrast to the case of microfibers spun in ammonia, whose crystallinity was not reduced. Note that the concentration was the same as that of NaOH (0.5 M) but the pH was lower (11.5) compared to that of NaOH (13.7). In addition, upon heat treatment, pure ChNF should have a maximum decomposition rate (*T*_dmax_) at 390 °C (marked as “2” in Fig. 3b). However, along with the *T*_dmax_ of wet-spun microfibers at 392 °C, there is a small peak at 300 °C (DTG, marked as “1” in Fig. 3b), which is associated with the decomposition of chitosan (deacetylation degree of about 50%).^39^ This means that some chitin was converted into chitosan during ChNF isolation. F_Na_ showed a higher peak at 300 °C than that of F_AM_ and F_AC_. Most likely, the use of 0.5 M NaOH coagulant partially deacetylated chitin into chitosan. The thermostability of the obtained microfibers is further discussed in the ESI, Fig. S6.†

**Fig. 4:**
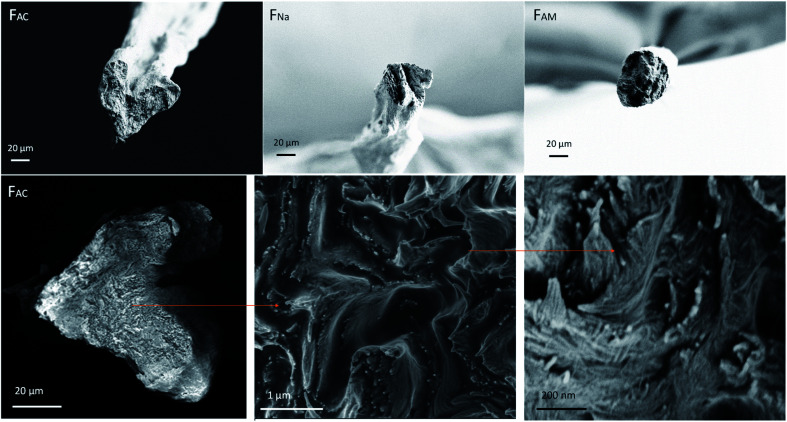
SEM images of cross-section at break (upper row) and a hierarchical structure observed from the cross-section of F_AC_ (bottom row).

Fig. 4 illustrates that the coagulants led to microfibers of different cross-section. Compared with F_Na_ and F_AM_, F_AC_ was irregular; however, this is likely the results of an artifact, as discussed for CNF systems:^34,40^ That is, the microfibers coagulated in alkaline solution, F_Na_ and F_AM,_ where free to form in the coagulation bath, with no influence of the bottom solid surface in the bath, which otherwise would have flattened the microfiber. Similar behavior was observed between acetone and CNF microfibers coagulated in electrolyte solutions.^34^

Besides the differences observed at the macroscale, all the spun microfibers possessed a hierarchical structure. Using F_AC_ as an example, ChNF assembled randomly and formed several fibrous units, which further formed into layers. These layers were packed *via* coagulation and drying. In addition, the layered structure was more noticeable at the circumference, likely induced by shear. Fig. S7† further confirms that nanofibrils tended to form layers and aligned in the lateral direction. A similar structure was found for microfibers obtained after polyelectrolyte complexation of TOCNF with chitosan as well as alginate with ChNF.^13,41^

### Mechanical properties

**Fig. 5:**
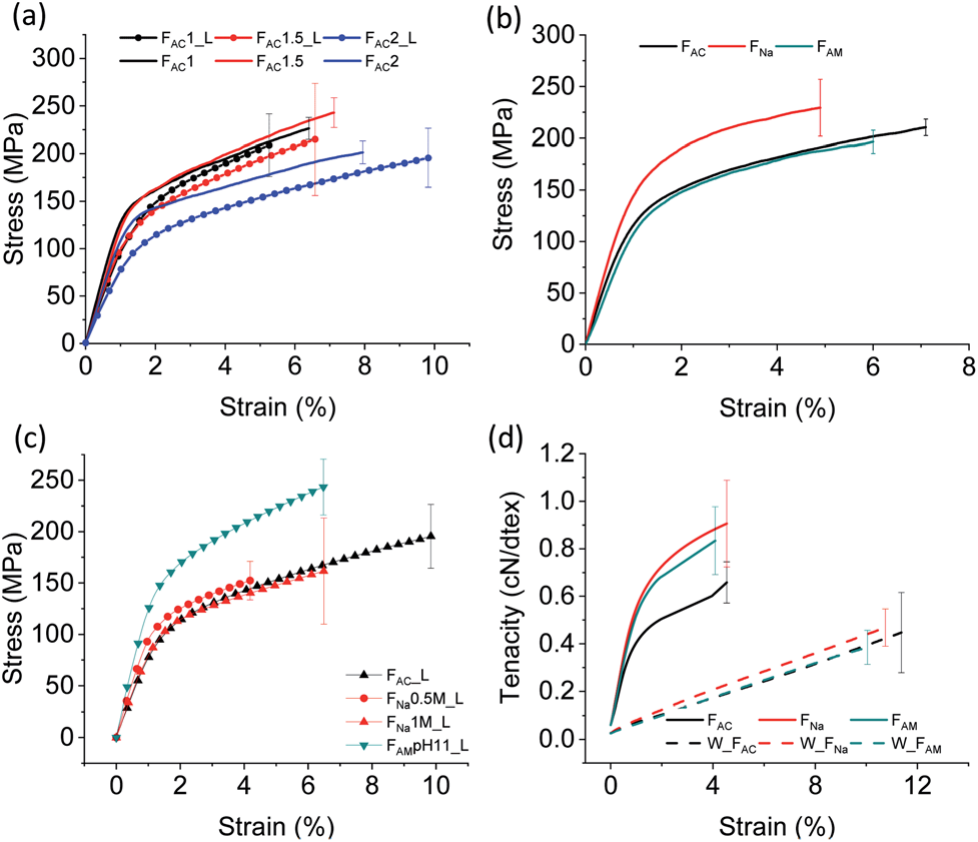
Mechanical properties of wet-spun microfibers: (a) ChNF with a solid content of 1, 1.5 and 2 wt% extruded in acetone at 1 m min^−1^ (line and symbol) and 2.6 m min^−1^ (solid line); (b) 2 wt% ChNF wet spun in acetone, 0.5 M NaOH and 0.5 M ammonia at 2.6 m min^−1^ (c) 2 wt% ChNF coagulated in alkaline solutions of different concentration at 1 m min^−1^. (d) Tenacity of microfibers coagulated from acetone, 0.5 M NaOH and 0.5 M ammonia at 2.6 m min^−1^.

The mechanical strength determines whether the microfibers sustain the demands of handling and processing, for example, as fibrous biomaterials and sutures. As such, the tensile strength of the spun microfibers was measured at 50% RH. We focused on the microfibers obtained from ChNF of 1, 1.5 and 2 wt% solid content and wet spun into acetone using two different extrusion rates: 1 m min^−1^ (_L) and 2.6 m min^−1^. As shown in Fig. 5a, similar values of tensile strength at break were observed for the microfibers, while a clear difference was noticed for the Young's modulus. F_AC_1_L and F_AC_1.5_L showed similar Young's modulus (11 GPa), higher than that of F_AC_2_L (8.5 GP). The trend was again observed after increasing the spinning rate: 14 GPa for F_AC_1 and F_AC_1.5 compared to 11 GPa for F_AC_2. It can be concluded that a dope of relatively low solids content and subjected to high shear leads to microfibers with higher Young's modulus, given the better fibril orientation.^42^

Table S1† summarizes the mechanical properties of the microfibers. The tensile strength and Young's modulus of the microfibers obtained here are significantly higher compared to those reported for ChNF microfiber spun in THF (132 MPa and 5 GPa, respectively).^12^ These values are even comparable to the microfibers spun at a 30% drawing ratio (223 MPa and 12.6 GPa).^12^ As the concentration increased, from 1 to 2%, the strain at break increased from 6.4% to 7.9%. It should be mentioned that even though F_AC_1 and F_AC_1.5 had a higher Young's modulus compared to that of F_AC_2, the microfibers were more prone to stick to the bottom of the bath container, which resulted in damage during collection. Hence, the dope with 2 wt% concentration was applied for alkaline coagulation and used in the remaining characterization.

Compared to those from 0.5 M ammonia, the microfibers coagulated in 0.5 M NaOH resulted in a higher Young's modulus and tensile strength (Fig. 5b). However, the strength was not affected significantly by increasing the NaOH concentration to 1 M (Fig. 5c). Under a reduced NaOH concentration (pH 11, similar to that of ammonia), microfibers could not be coagulated. Microfibers coagulated in ammonia (pH 11) presented much higher mechanical properties than those coagulated in acetone and NaOH. The difference may be related to the smaller cross-sectional area: 0.0029 mm^2^ for F_AM_ compared to 0.0051 mm^2^ for microfibers coagulated in 0.5 M NaOH and 0.0043 mm^2^ for those in 1 M NaOH.

### Water and moisture sorption

**Fig. 6:**
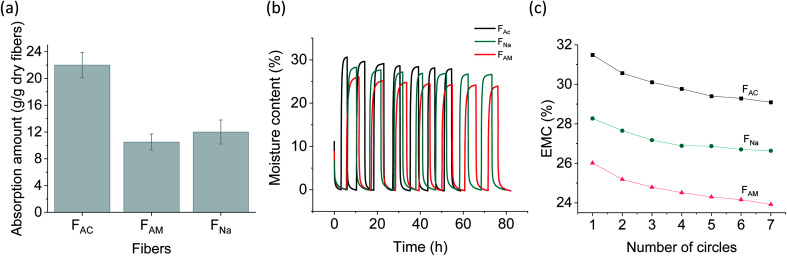
Water and moisture influence on wet-spun microfibers: (a) water sorption capacity. (b) Dynamic vapor sorption (DVS) analysis with RH cycles between 0% and 95% (seven cycles). (c) Evolution of the equilibrium moisture content at RH 95% for 7 cycles.

Water and moisture sorption correlate with the “capillarity” (the extent to which absorbed fluid is transferred along the microfibers) and “fluid absorption” (ability to take up fluid after immersion) of fibrous biomaterials. When immersed in water, the microfibers swelled due to diffusion and absorption in the inter-spaces between fibrils. Microscope images (Fig. S8†) show that F_AC_ swelled significantly, reaching a diameter over three times higher than that of the dry microfibers (312 *versus* 90 μm). The relative increase in diameter is more marked compared to that of F_Na_ (∼1.2 times) and F_AM_ (∼1.7 times). Water sorption capacity was also measured and found to be consistent with the morphological observations (Fig. 6a). The water sorption capacity of F_AC_ (22 g g^−1^) was significantly higher than that of F_AM_ (10.5 g g^−1^) and F_Na_ (12 g g^−1^). The higher water sorption of F_AC_ is likely the results of the higher surface charge of the fibrils. In addition, compared to F_AM_, some residual sodium ions might remain in F_Na,_ resulting in a slightly higher water binding. Interestingly, F_AC_ possessed significantly higher water sorption than the acetone-coagulated CNF microfibers (1.1 g g^−1^),^9^ though the partially deacetylated ChNF was supposed to be more hydrophobic. This observation is likely related to the microfiber structure: the more hydrophilic CNF collapsed in the organic solvent, resulting in a relatively more dense structure.^43^ Furthermore, the water absorption capacity of F_AC_ was much higher than that of typical commercial absorbent fluff pulp (12 g g^−1^),^44^ opening possible uses as renewable absorbents.

The microfibers were further exposed to air under cyclic relative humidity (RH), between 0% and 95% (7 cycles, Fig. 6b). A similar behavior was observed for all microfiber types, unlike water sorption observations. Moisture sorption at an equilibrium moisture content of 95% followed the trend: F_AC_ (31%) > F_Na_ (28%) > F_AM_ (26%). After seven RH cycles, a progressive decline in moisture sorption capability occurred (Fig. 6c), which is most likely the result of the “hornification” phenomenon, *e.g.*, from the irreversible hydrogen bonding formation, pore closure and fibril aggregation.^45–47^

### Biocompatibility and cell proliferation

**Fig. 7:**
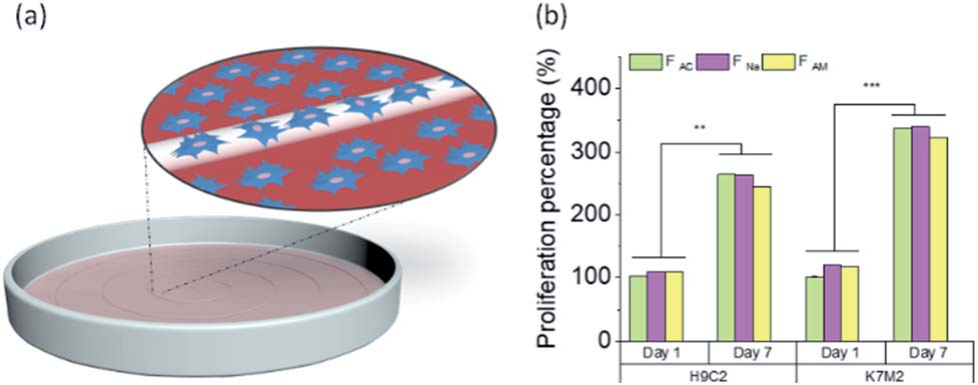
(a) Schematic illustration of cell proliferation. (b) Cell proliferation percentage based on positive control on first time point of H9c2 (cardiac myoblast) and K7M2 (bone osteoblast) in the vicinity of wet-spun microfibers coagulated in different baths (acetone, NaOH, and ammonia solution) (*n* = 4) during 1 week at 37 °C in 5% CO_2_. One-way analysis of variance (ANOVA) was used for statistical analysis with the level of significance set at probabilities of **p < 0.01 and ***p < 0.001.

We investigated whether the microfibers are biocompatible or if they show any sign of toxicity to given cell lines. Here, wet-spun chitin microfibers obtained from the different coagulants were investigated in terms of biocompatibility, where the cell interaction with chitin microfibers was examined and compared with the control (without microfibers) (Fig. 7a).

The cell proliferation of rat cardiac myoblast H9c2 and mouse bone osteoblast K7M2 in the vicinity of the chitin microfibers were tested during one week. Fig. 7b shows the cell proliferation of more than twice for both cell types, compared to the positive control in the first time point, with high viability, Fig. S9,† demonstrating that all three types of chitin microfibers are biocompatible. The data supports the experimental evidence that if some trace of the coagulants remained in the sample, they did not interfere with the proliferation of the cells, even after one week contact between the biomaterial with the cells.

## Conclusions

Partially deacetylated chitin nanofibrils were processed into microfibers by using wet-spinning with different coagulants (organic and alkaline solvents). A hierarchical structure was observed in all microfibers, with fibrils aligned in the axial direction. The SEM observations and the low crystallinity obtained by WAXS revealed that F_Na_ was partially dissolved and regenerated during NaOH coagulation. The spinning dope of low solids content and processed at high rates produced microfibers of higher stiffness. The microfibers obtained in alkaline solution displayed similar mechanical properties compared to those obtained in the organic solvent (acetone). After immersion in water, all the microfibers presented a high water sorption capacity and retained their strength (∼50% of the dry strength). The cell proliferation studies with rat cardiac myoblast H9c2 and mouse bone osteoblast K7M2 in the vicinity of the chitin microfibers successfully demonstrated that cells proliferate significantly when in contact with the microfibers, even after one-week contact. Overall, this study provides the initial steps for further development of fibrous bioproducts in the form of single microfibers or as woven/non-woven patches for potential applications in heart and bone tissue engineering.

## Conflicts of interest

There are no conflicts to declare.

## Supplementary Material

RA-010-D0RA06178F-s001
