# Supplementary material for: Microfibers synthesized by wet-spinning of chitin nanomaterials: mechanical, structural and cell proliferation properties
Source: RSC Adv. 2020 Aug 10;10(49):29450–9. doi: 10.1039/d0ra06178f (PMC9059162; doi:10.1039/d0ra06178f)
Supplement: RA-010-D0RA06178F-s001 [file RA-010-D0RA06178F-s001.pdf]

Electronic Supplementary Material (ESI) for RSC Advances.
This journal is © The Royal Society of Chemistry 2020

Supporting Information

Microfibers Synthesized by Wet-Spinning of Chitin Nanomaterials: Mechanical, Structural and Cell Proliferation Properties

Ling Wang,[a] *Nazanin Zanjanizadeh Ezazi,*[b] *Liang Liu,c Rubina Ajdary,*[a] *Wenchao Xiang,*[a] *Maryam Borghei,*[a] *Hélder A. Santos,*[b,d] *Orlando J. Rojas*[a,e,*]

[a] *Department of Bioproducts and Biosystems, Aalto University, P.O. Box 16300, 00076 Aalto, Finland.*

[b] *Drug Research Program, Division of Pharmaceutical Chemistry and Technology, Faculty of Pharmacy, University of Helsinki, FI 00014, Helsinki, Finland.*

[c] *College of Chemical Engineering, Nanjing Forestry University, Nanjing, 210037, China.*

[d] *Helsinki Institute of Life Science (HiLIFE), University of Helsinki, FI 00014, Helsinki, Finland.*

[e] *Bioproducts Institute, Departments of Chemical and Biological Engineering, Chemistry and Wood Science, University of British Columbia, 2360 East Mall, Vancouver, BC Canada V6T 1Z3.*

Corresponding authors: O.J.R: orlando.rojas@ubc.ca

Number of pages: 9
Number of Tables: 1
Numbers of Figures: 9

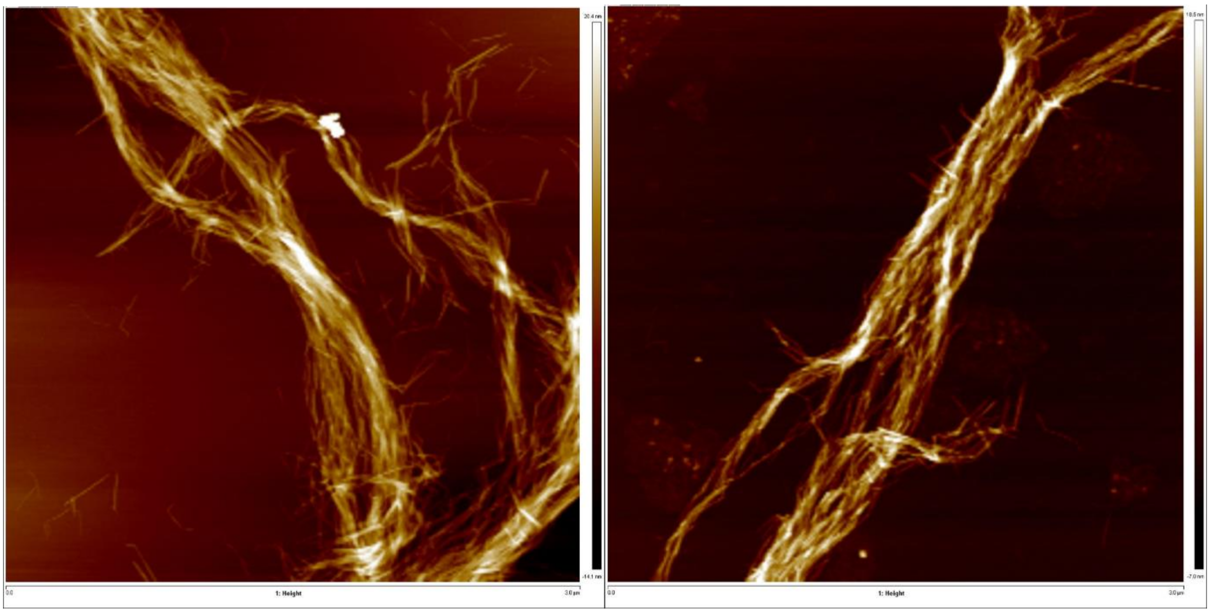

Fig. S1. AFM images (3 x 3 μm) of swollen ChNF nanofibrils.

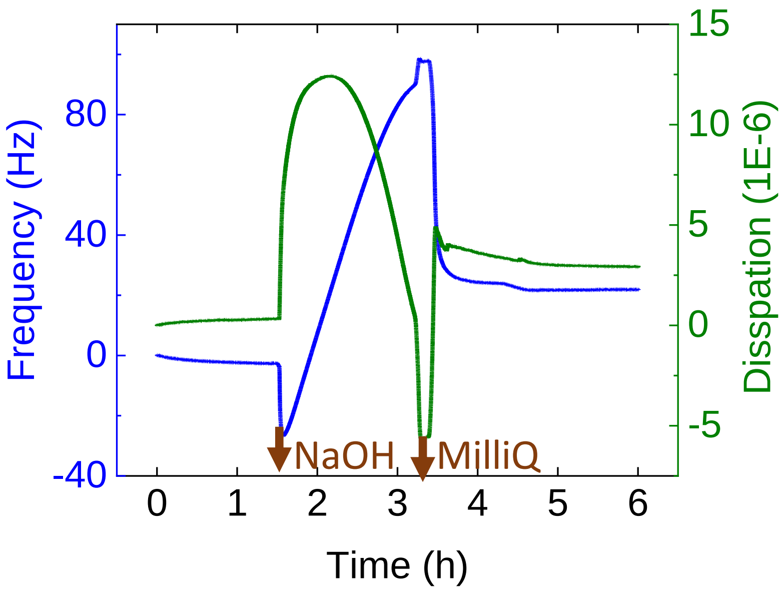

Fig. S2. Frequency and dissipation of ChNF-coated QCM-D crystals as a function of time upon contact with NaOH (no flow/ batch mode).

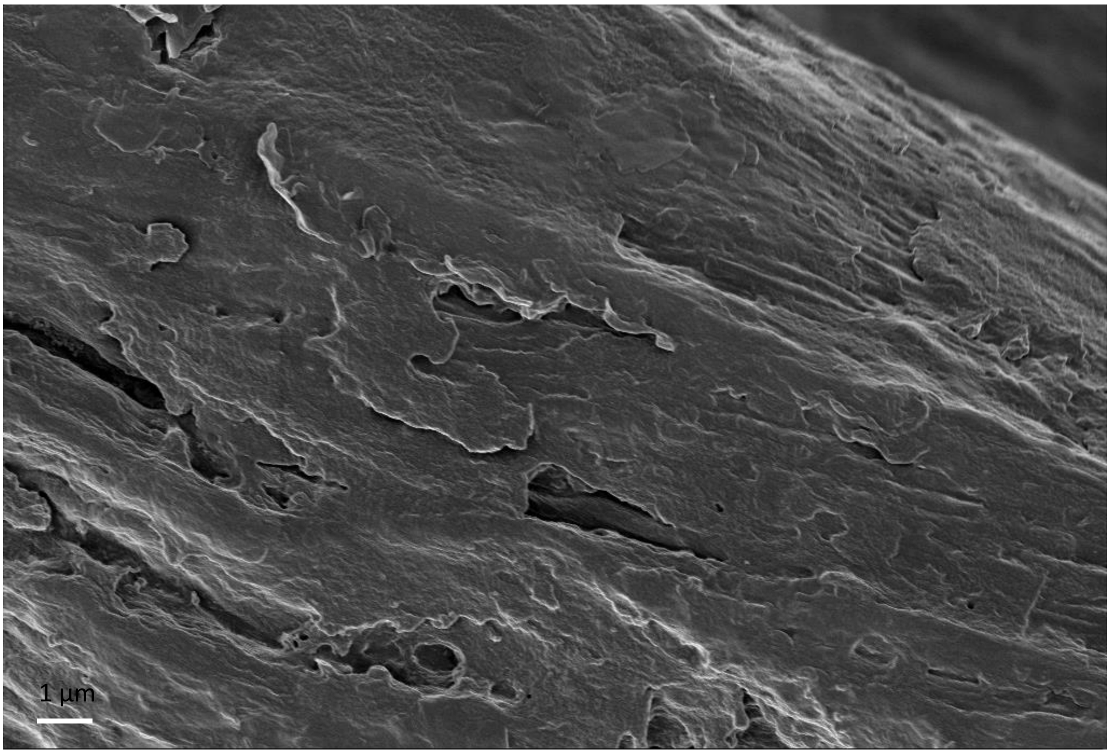

Fig. S3. The surface morphology of F_{Na}.

Fibril orientation. Wide Angle X-ray Scattering (WAXS) was applied to detect the ChNF nanocrystal orientation in spun filament. A MicroMax-007 X-ray generator (Rigaku, Japan) operated at a wavelength of 1.54 Å, with a beam size of 120 µm and exposure time of 10 minutes. A Mar345 plate detector was equipped to collect sample diffraction patterns with 200 mm distance of sample-to-detector. Before evaluation, the background was subtracted from the diffraction patterns. Based on azimuthal intensity distribution profiles, orientation index (π) and Herman's orientation parameter (S) were calculated according to equations equation (1) and (2).

$$\pi = \frac{180^{\circ} - FWHM}{180^{\circ}} \quad (1)$$

where FWHM is the full width at the half maximum (in degrees) of one of the two peaks in the azimuthal intensity distribution profile. π was calculated for both peaks and their average reported.

$$S = \frac{3}{2} < cos^2\gamma > - \frac{1}{2} \qquad (2)$$

Assuming cylindrical symmetry in the filament, the average cosine ‹cos²γ› was obtained from the azimuthal angle φ according to equation (3).[1]

$$\langle cos^2\gamma \rangle = 1 - 2\langle cos^2\varphi \rangle \qquad (3)$$

where

$$< cos^2\varphi >= \frac{\sum_{\varphi_0}^{\varphi_0+\pi/2} I(\varphi)\sin\varphi\, cos^2\varphi}{\sum_{\varphi_0}^{\varphi_0+\pi/2} I(\varphi)\sin\varphi}$$

Here, $I(\varphi)$ is the intensity detected at azimuthal angle φ, and φ_0 is the azimuthal angle in the beginning of the range used for the calculation of the average cosine $\langle cos^2\varphi \rangle$. S was calculated at φ_0 of 0, π/2, π and 3π/2 and the average of these values is reported. A value of 1 for the orientation parameter indicates a fully oriented structure while 0 means a disordered structure.

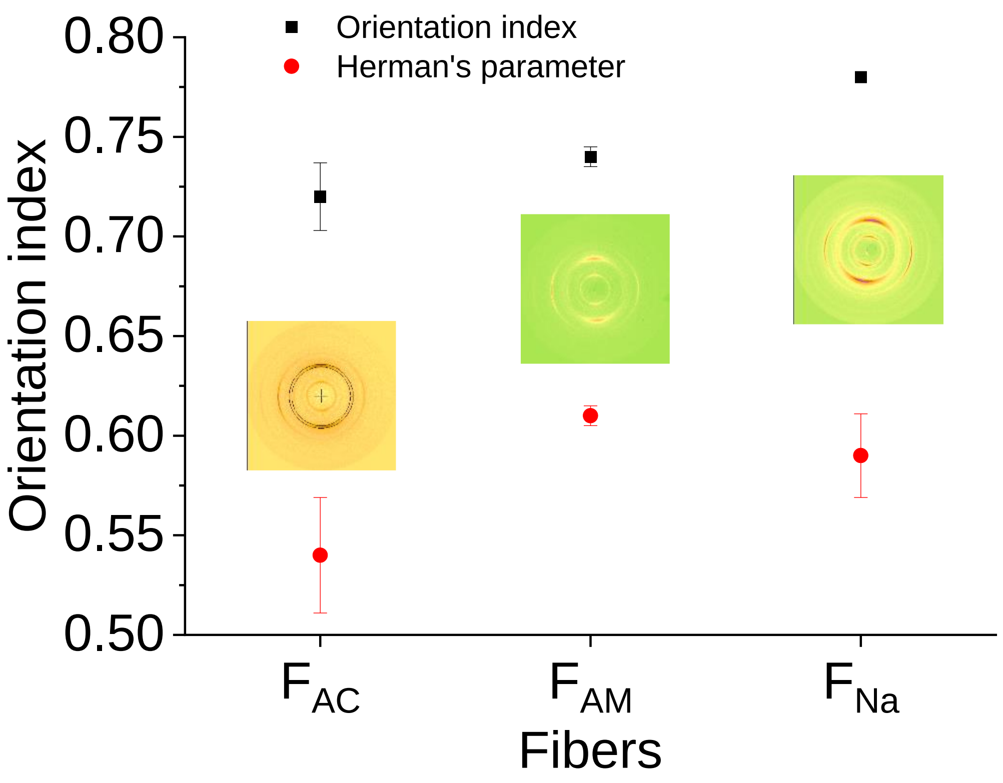

Fig. S4. fibril orientation degree in terms of orientation index and Herman's parameter. Note: insets are respective diffraction diagrams.

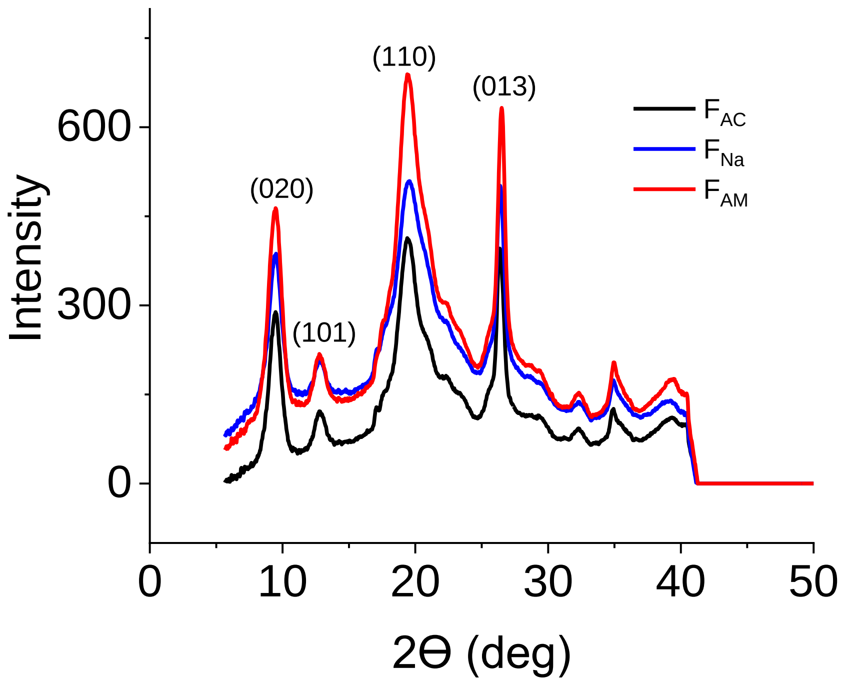

Fig. S5. WAXS diffraction diagrams.

Thermal stability. Thermogravimetric analysis (TGA) was utilized to analyze thermal stability of the spun filaments by measuring the weight change as a function of temperature in N_2 atmosphere (TA Instrument, Thermo Gravimetric Analyzer Q500). The filaments were cut into short pieces and heated up to 900 °C from room temperature with a heating rate of 10 °C min^{-1}.

Thermal stability of the spun filaments was determined by thermalgravimetric analysis, from room temperature to 900 °C under nitrogen (**Fig. S8**). All fibers possessed similar thermograms regarding to both TGA and DTG. There are two main weight losses indicating a three-stage polymer degradation via heat treatment: first one arounf 100 °C was attributed to water desorption, marked as 1 in **Fig. S8**, then within the temperature range of 200-400 °C, the saccharide structure was degraded starting with the ChNF of lower degree of polymerization (marked as 2 in **Fig. S8**). F_{Na} started to degrade (T_{onset}) at higher temperature (286 °C) than F_{AC} and F_{AM} (263 °C). Meanwhile. according to the peak at 300 °C in DTG (marked as 3 in **Fig.**

S8), some chitin might be transformed to chitosan (with the deacetylation degree of reaches about 50%).[2] In addition, F_{Na} illustrated higher peak at 300 °C than that of F_{AM} and F_{AC}. Most likely, 0.5 M NaOH solution as coagulants were partially deacetylate chitin into chitosan. The maximum decomposition rate (Td_{max}) of ChNF occurred at 392 °C (marked as 4 in **Fig. S8**), which is much higher than the spun fibers made from CNF (308 °C) and TOCNF (261 °C).[3,4] Finally, by raising the temperature above 400 °C, complete decomposition and carbonization occurred (marked as 5 in **Fig. S8**). It can also be seen that a higher mass yield was obtained from F_{AC} and F_{Na} (30%) compared to F_{AM} (23%). The values were higher than the reported mass residues from wet-spun TOCNF fibers (4%-24%).[4,5] The mass yield can be increased by an optimized temperature profile during carbonization.[5–7]

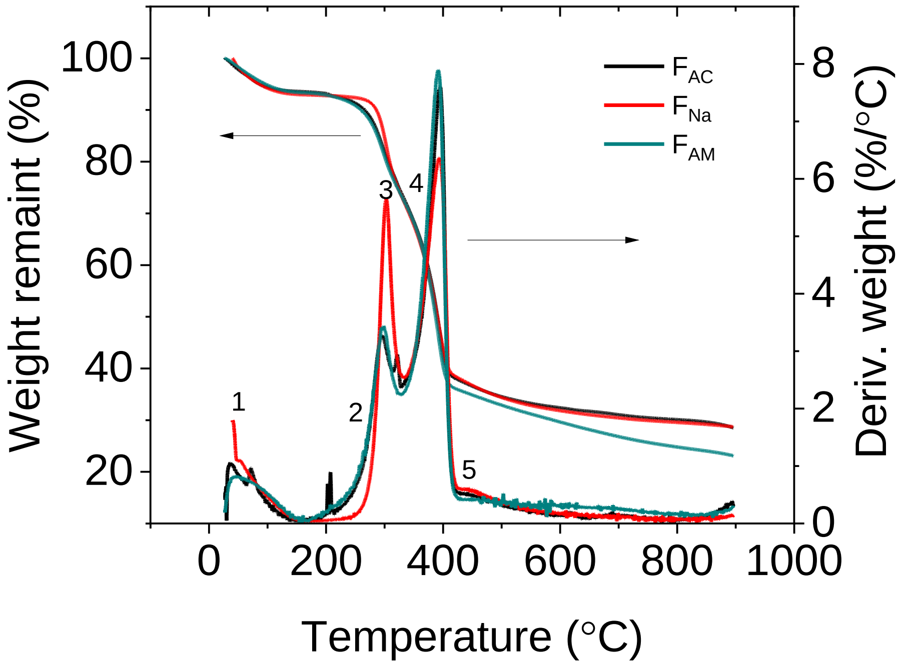

Fig. S6. Thermogravimetry analysis (TGA) and differential thermogravimetric (DTG) profiles of wet-spun ChNF fibers from different coagulants.

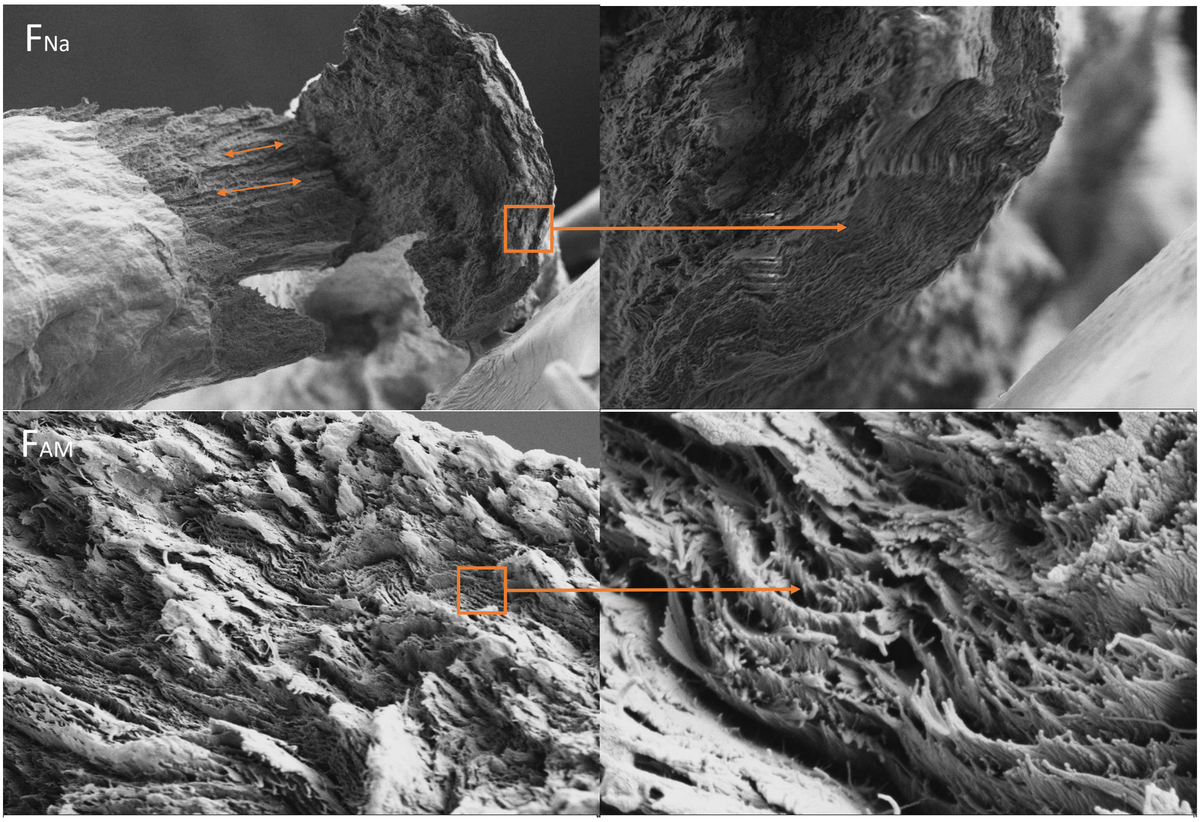

Fig. S7. The SEM images of cross-section at break from F_{Na} and F_{AM}.

Table S1. Mechanical properties of wet spun microfibers.

**Fibers**	**Young's modulus/GPa**	**Tensile strength /MPa**	**Strain at break /%**
F_{AC}1_L	11.8±4.2	234±32.7	5.7±2.2
F_{AC}1.5_L	11.2±2.9	216±58.7	6.6±1.5
F_{AC}2.2_L	8.5±1.2	193±30.7	9.8±2.5
F_{AC}1	13.7±2.6	231.4±47.9	6.4±2.6
F_{AC}1.5	14.1±2.4	245.8±39.9	7.1±3.8
F_{AC}2.2	11.4±2.2	194.4±38.5	7.9±1.7
F_{Na}0.5M	13.5±2.9	233.4±57.3	7.5±1
F_{AM}0.5M	8.8±0.8	187.6±35.6	10.6±3.1
F_{Na}0.5M_L	11.1±0.8	151±18.7	4.2±0.8
F_{Na}1M_L	8.9±1.2	164±51.6	6.5±2
F_{AM}pH11_L	13.6±2.2	217±27.2	5.9±1.9

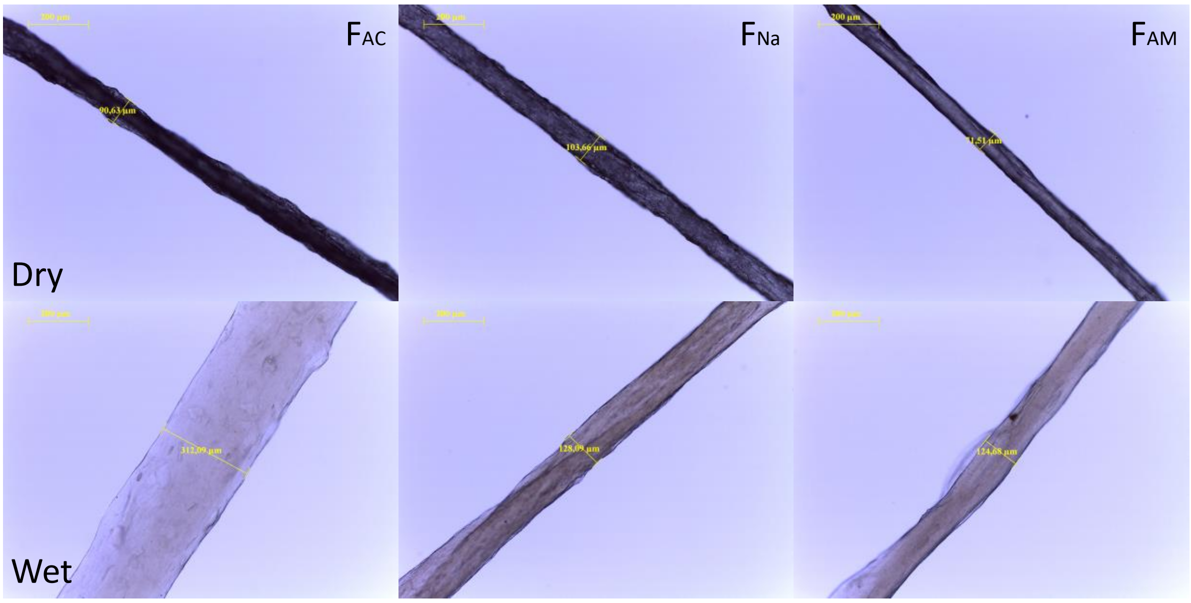

Fig. S8. Optical microscope images of ChNF fibers in dry (upper row) and wet (bottom row) state.

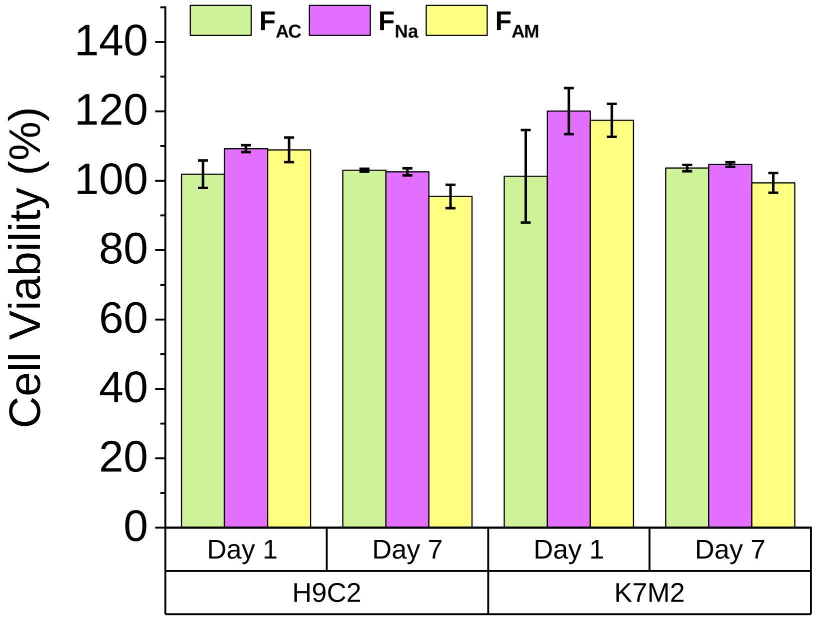

Fig. S9. Effect of wet spun fibers coagulated in different coagulants on H9c2 (cardiac myoblast) and K7M2 (bone osteoblast) viability (n=4). Both cells stayed viable during 7 days of experiment.

References

(1) Nishiyama, Y.; Kuga, S.; Wada, M.; Okano, T. Cellulose Microcrystal Film of High Uniaxial Orientation. *Macromolecules* **1997**, *30* (20), 6395–6397. https://doi.org/10.1021/ma970503y.

(2) S. Dassanayake, R.; Acharya, S.; Abidi, N. Biopolymer-Based Materials from Polysaccharides: Properties, Processing, Characterization and Sorption Applications. In *Advanced Sorption Process Applications*; IntechOpen, 2019. https://doi.org/10.5772/intechopen.80898.

(3) Cunha, A. G.; Lundahl, M.; Ansari, M. F.; Johansson, L. S.; Campbell, J. M.; Rojas, O. J. Surface Structuring and Water Interactions of Nanocellulose Filaments Modified with Organosilanes toward Wearable Materials. *ACS Applied Nano Materials* **2018**, *1* (9), 5279–5288. https://doi.org/10.1021/acsanm.8b01268.

(4) Wang, L.; Lundahl, M. J.; Greca, L. G.; Papageorgiou, A. C.; Borghei, M.; Rojas, O. J. Effects of Non-Solvents and Electrolytes on the Formation and Properties of Cellulose I Filaments. *Scientific Reports* **2019**, *9* (1), 1–11. https://doi.org/10.1038/s41598-019-53215-0.

(5) Wang, L.; Ago, M.; Borghei, M.; Ishaq, A.; Papageorgiou, A.; Lundahl, M. J.; Rojas, O. J. Conductive Carbon Microfibers Derived from Wet-Spun Lignin/Nanocellulose Hydrogels. *ACS Sustainable Chemistry & Engineering* **2019**, acssuschemeng.8b06081. https://doi.org/10.1021/acssuschemeng.8b06081.

(6) Huang, X. Fabrication and Properties of Carbon Fibers. *Materials* **2009**, *2* (4), 2369–2403. https://doi.org/10.3390/ma2042369.

(7) Wang, L.; Borghei, M.; Ishfaq, A.; Lahtinen, P.; Ago, M.; Papageorgiou, A. C.; Lundahl, M. J.; Johansson, L.-S.; Kallio, T.; Rojas, O. J. Mesoporous Carbon Microfibers for Electroactive Materials Derived from Lignocellulose Nanofibrils. *ACS Sustainable Chemistry & Engineering* **2020**. https://doi.org/10.1021/acssuschemeng.0c00764.
